# Investigation of Different Molecular Weight Fucoidan Fractions Derived from New Zealand *Undaria pinnatifida* in Combination with GroA Therapy in Prostate Cancer Cell Lines

**DOI:** 10.3390/md16110454

**Published:** 2018-11-18

**Authors:** Xu Yang, Sheng Wang, Sari Schokoroy Trangle, Yan Li, William Lindsey White, Jinyao Li, Tianlei Ying, Qingjun Kong, Yu Zhao, Jun Lu

**Affiliations:** 1 School of Science, Faculty of Health and Environmental Sciences, Auckland University of Technology, Auckland 1010, New Zealand; cindy.yang@aut.ac.nz (X.Y.); kelvin.wang@aut.ac.nz (S.W.); yan.li@aut.ac.nz (Y.L.); lindsey.white@aut.ac.nz (W.L.W.); 2 Department of Neurobiology, Tel-Aviv University, Ramat-Aviv 69978, Israel; sari.trangle@gmail.com; 3 School of Interprofessional Health Studies, Faculty of Health and Environmental Sciences, Auckland University of Technology, Auckland 1010, New Zealand; 4 Xinjiang Key Laboratory of Biological Resources and Genetic Engineering, College of Life Science and Technology, Xinjiang University, Urumqi 830046, Xinjiang, China; ljyxju@xju.edu.cn; 5 Key Laboratory of Medical Molecular Virology of MOE/MOH, Shanghai Medical College, Fudan University, 130 Dong An Road, Shanghai 200032, China; tlying@fudan.edu.cn; 6 College of Food Engineering and Nutritional Science, Shaanxi Normal University, Xi’an 710119, Shaanxi, China; kongqingjun1976@snnu.edu.cn; 7 College of Life Sciences, Shanghai Normal University, 100 Guilin Road, Shanghai 200234, China; zhaoyu@shnu.edu.cn; 8 Institute of Biomedical Technology, Auckland University of Technology, Auckland 1010, New Zealand; 9 College of Life Sciences and Oceanography, Shenzhen University, Shenzhen 518071, China

**Keywords:** fucoidan, low molecular weight fucoidan, Nucleolin, GroA, prostate cancer, *Undaria pinnatifida*

## Abstract

Fucoidan, a sulfated polysaccharide extracted from brown seaweeds, has been shown to possess various antioxidant, anticoagulant, antiviral, and anticancer functions. In this study, we focused on low molecular weight fucoidan (LMWF) which was extracted from New Zealand *Undaria pinnatifida*, and investigated its anti-proliferative effects, combined with a quadruplex-forming oligonucleotide aptamer (GroA, AS1411), a powerful cell surface Nucleolin inhibitor, in prostate cancer cells. We examined LMWF (<10 kDa) and compared it with laboratory grade Fucoidan purchased from Sigma (FS), all extracted from the same seaweed species *U. pinnatifida*. We found that LMWF significantly improved the anti-proliferative effect of GroA, as it decreased cancer cell growth and viability and increased cell death. This research may provide the foundation for LMWF to be used against prostate cancers as a supplement therapy in combination with other therapeutic agents.

## 1. Introduction

Fucoidan, fucose-containing sulphated polysaccharides, is the term commonly used to describe water-soluble compounds extracted from brown seaweeds. Despite the diversity in their structure and composition, these polysaccharides have some structural features in common [1]. Fucoidan is mainly found in the cell wall and intercellular space of brown seaweeds [2], where it can make up to over 40% dry weight of the seaweed cell walls [3] and up to 16% dry weight of the whole alga [2].

Fucoidan has numerous proven bioactivities, such as antioxidant [3], anticoagulant [4], antiviral [5], and anticancer functions 8. These bioactivities are linked to the molecular weight [6], composition (e.g., monosaccharide composition, the degree of sulphation) [7] and structure [8]. However, it is known that Fucoidan varies significantly between the source species, the environment, the source of which the seaweeds were cultivated in or harvested from, and even the time of the year in which it is harvested [9]. No two isolated Fucoidans are exactly the same, even if they are extracted from the same seaweed species; they are all unique in their structure, composition, and bioactivities [10].

Fucoidan is likely to be used as a supplemental therapy for cancer treatment, where there are known effective therapies [11,12]. Molecular weight is a crucial factor in Fucoidan activity. Yang et al. have suggested that the anticancer activity of Fucoidans could be significantly improved by lowering their molecular weight when they are depolymerized by mild hydrolysis conditions without causing considerable desulfation [13]. Lee’s group also reported that Low molecular weight Fucoidan attenuates the growth of human prostate cancer cells both in vitro and in vivo [14].

Fucoidan’s anticancer activity is mainly due to its pro-apoptotic [15,16] and antiangiogenic activities [17,18]. Fucoidan may find a role either to reduce side effects, to enhance the therapeutic effects of conventional therapy, or to address cancers for which there are no known therapy options. Current literature has suggested additive or synergistic activity of Fucoidan in combination with chemotherapy agents to improve clinical outcomes. The Epidermal Growth Factor Receptor (EGRF; ErbB-1) is a member of the ErbB family of receptors, whose mutations have been identified as affecting ErbB-1 expression or activity and are associated with cancer. The effect of fucoidan on the EGF-induced phosphorylation of ErbB-1 was tested by Lee et al. [19]; results indicated that treatment with fucoidan significantly decreased the phosphorylation of EGFR (ErbB-1), but not the EGFR (ErbB-1) total protein level. Zhang and colleagues [20] studied the therapeutic efficiency of Fucoidan in combination with cisplatin, tamoxifen, and paclitaxel, and their findings showed an improvement in cell proliferation reduction in the MCF-7 cell line. Another study by Atashrazm et al. [21] determined the synergistic potential of Fucoidan with lapatinib, and results showed a reduction in the cell growth of a breast cancer cell line, OE33, through activity comparable to tyrosine kinase inhibitors. Recently, Thakur et al. [22] observed a strong synergy between lapatinib and Fucoidan, as the addition of Fucoidan increased the anti-tumor properties of lapatinib, and importantly, improved the test animal’s well-being.

Nucleolin inhibitor GroA (AS1411) is a first-in-class anticancer agent, currently in Phase II clinical trials, which is a 26-base guanine-rich oligonucleotide (GRO) with an unmodified (phosphodiester) DNA backbone [23]. This molecule and its related analogs can inhibit proliferation and induce cell death in many types of cancer cells, but have little effect on normal cells [24]. Previous experiments show that antiproliferative GROs such as AS1411 can build stable G-quadruplex structures and produce an unusual resistance to cellular and serum nucleases. Moreover, GROs can bind directly and selectively to Nucleolin, and the growth inhibitory activity of GROs is positively correlated with their ability to bind this protein [24]. Additionally, several biological effects of AS1411 have been shown to result from its ability to alter the subcellular localization of certain Nucleolin-containing complexes, or to interfere with the molecular interactions of Nucleolin [23].

Ronit Pinkas-Kramarski’s lab in Israel has identified non-nucleolar Nucleolin as an ErbB receptor-interacting protein [25,26]. This interaction leads to receptor dimerization and activation, as well as to increased colony growth in soft agar. Previous studies have also identified a crosstalk between Nucleolin, ErbB1, and Ras proteins, and demonstrated that treatment with a combination of FTS (which inhibits the Ras proteins) and GroA (AS1411, which targets cell surface Nucleolin) has a stronger inhibitory effect on several types of cancer cells, i.e., colon cancer cells (DLD-1 and HCT-116), prostate cancer cells (DU-145 and PC-3) and glioblastoma cells (U-87), leading to the inhibition of cell growth and anchorage independent growth [27,28,29]. Therefore, it seems likely that combining two drugs, Fucoidan and GroA, may have a better inhibitory effect on ErbB-1 receptor activation, and from that activity, a stronger inhibitory effect on cancer cell growth and tumorigenicity.

The aim of this study is to determine whether Low-molecular-weight Fucoidan (LMWF) and Sigma Fucoidan (FS), combined with GroA, will synergize in inhibition of prostate cancer cells. We hypothesize that this combination of drugs may have a better inhibitory effect on ErbB-1 receptor activation, and subsequently, a stronger effect on prostate cancer cell growth and viability. We compared the proliferative effect of LMWF and FS, both of which are isolated from *Undaria pinnatifida*, in two lines of prostate cancer cells, and comment on the Fucoidans’ possible mechanism of action.

## 2. Results and Discussion

### 2.1. Comparison of Inhibitory Effects between Types of Fucoidan Combined with GroA

Based on previous studies, different LMWF concentrations (100, 200 and 300 µg/mL) and different FS concentrations (500, 750 and 1000 µg/mL) were prepared to treat PC-3 and DU-145 cells. LMWF and FS both reduced cell growth and viability in a dose- dependent manner at 72 h and 96 h time points (Supplement Figures S1 and S2). Control experiments did not show any effect. As shown in the PC-3 cell line, cell viability was higher with FS compared to LMWF concentrations at 100, 200 and 300 µg/mL (Supplement Figure S1). There were also no significant differences in cell viability (*p* > 0.05) between the three different FS concentrations. Cell viability at 72 h was 85–90%, and at 96 h was approximately 82%. DU-145 cell viability showed the same tendency (Supplement Figure S2). Alternatively, LMWF (300 µg/mL) treatments showed that cell viability was remarkably decreased after 72 h and 96 h, in both the PC-3 and DU-145 cell lines. The decrease in cell viability is also shown at 100 and 200 µg/mL of LMWF.

Next, we sought to examine the combined effect of LMWF and FS with GroA, a powerful Nucleolin inhibitor, on cancer cell growth and viability at lower concentration range. The optimal concentration of GroA used in this study was 10 µM, based on a previous study and Cro, as the inactive oligomer form [29]. For both cell lines, GroA/Cro concentrations were consistent at 10 µM, whereas LMWF concentrations were 100, 220, and 300 µg/mL, and FS concentrations were 500, 750, and 1000 µg/mL.

As shown in supplement Figures S3 and S4, when cells were treated for 72 h with LMWF alone, cell viability was reduced to 53–63%. When cells were treated with GroA alone, cell viability was reduced to about 60%. However, following co-treatment with GroA combined with 100, 220, or 300 µg/mL LMWF, cell viability was significantly lower, compared to each of the treatments alone. Similarly, the combined treatment had an enhanced inhibitory effect after 96 h of treatment, compared with each of the treatments alone, as the number of viable cells was under 50%. Similar results were obtained in the DU-145 cell line. In all groups tested, increasing the concentration of LMWF combined with GroA resulted in the gradual reduction of cell growth. Therefore, LMWF was able to enhance the inhibitory effect of GroA on cell growth of PC-3 and DU-145 cells in a dose- dependent manner, even at low concentrations.

The combined treatment of FS with GroA resulted in a different outcome compared to the LMWF combination treatments (Supplement Figures S5 and S6). The number of viable cells following treatments with 500, 750, and 1000 µg/mL FS did not show a remarkable inhibitory effect (10%), as compared to the control. Cell viability following treatment with GroA for 72 h was about 60%. However, when PC-3 cells were co-treated with GroA and FS, there was no significant difference compared with GroA-alone. DU-145 cell line showed similar results in all the groups that were tested. In all groups, despite increasing the concentration of FS, only GroA decreased the cell viability of both cell lines. Hence, FS was not able to enhance the inhibitory effect of GroA on cell growth and viability of PC-3 and DU-145 cells in a dose-dependent manner, even at a very high concentration range.

**Figure 1:**
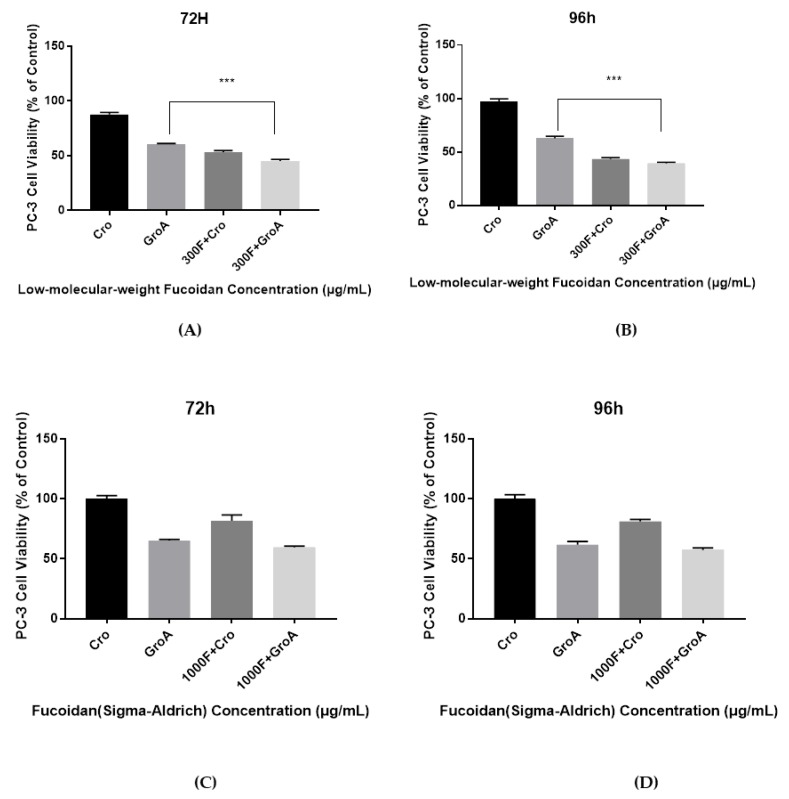
The Combined inhibitory effect of GroA/Cro and LMWF (**A**,**B**), or in combination with FS (**C**,**D**) on the growth of PC-3 cells after an incubation time of 72 h (**A**,**C**) or 96 h (**B**,**D**). Cells were incubated in the presence of various concentrations of LMWF (300 µg/mL), GroA/Cro (10 µM), and compared with combination treatments (LMWF + GroA/Cro) (**A**,**B**). Cells were incubated in the presence of various concentrations of FS (1000 µg/mL), GroA/Cro (10 µM), and compared with combination treatment (FS + GroA/Cro) (**C**,**D**). A relative cell viability of 100% was designated as the total number of cells that grew after 72 and 96 h cultures in the absence of LMWF and GroA/Cro. Data is presented as means ± S.D., n = 6. Asterisks indicate a value significantly different, *** *p* < 0.001 (Student’s *t*-test).

**Figure 2:**
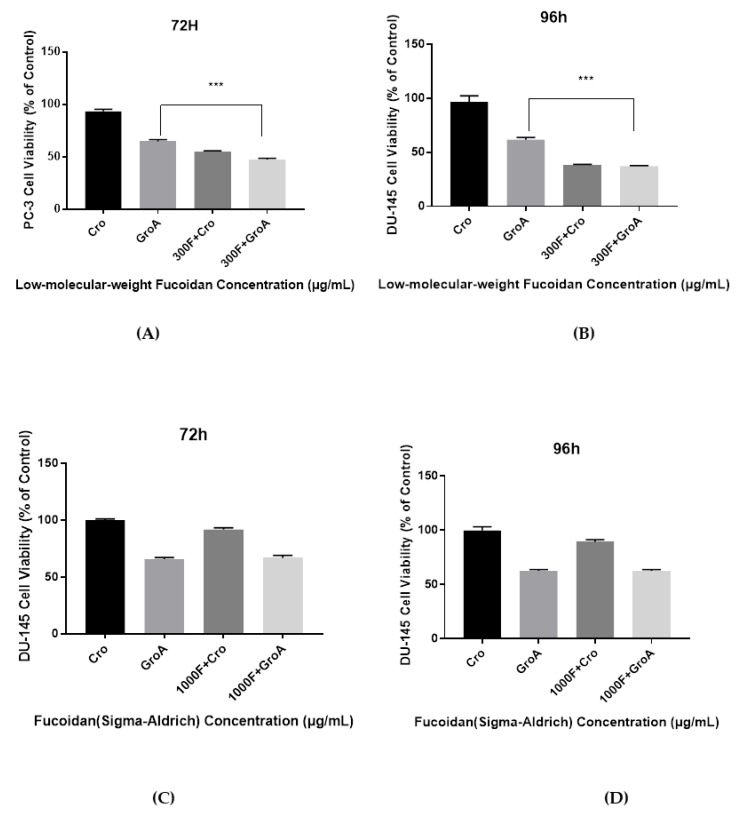
The Combined inhibitory effect of GroA/Cro and LMWF (**A**,**B**), or in combination with FS (**C**,**D**) on the growth of DU-145 cells after an incubation time of 72 h (A, C) or 96 h (**B**,**D**). Cells were incubated in the presence of various concentrations of LMWF (300 µg/mL), GroA/Cro (10 µM), and compared with combination treatments (LMWF + GroA/Cro) (**A**,**B**). Cells were incubated in the presence of various concentrations of FS (1000 µg/mL), GroA/Cro (10 µM), and compared with combination treatment (FS + GroA/Cro) (**C**,**D**). A relative cell viability of 100% was designated as the total number of cells that grew after 72 and 96 h cultures in the absence of LMWF and GroA/Cro. Data is presented as means ± S.D., n = 6. Asterisks indicate a value significantly different, *** *p* < 0.001 (Student’s *t*-test).

The cell viability results demonstrate that the combined effect of LMWF and GroA on the reduction of PC-3 and DU-145 cells’ growth was significantly higher than with either LMWF (100, 220 and 300 µg/mL) or GroA-alone (10 µM). However, the results of FS combined with GroA, indicated that only a GroA-related effect could be observed (Figure 1 and Figure 2). A possible explanation for this observation might be that FS at the concentration of 500, 750, and 1000 µg/mL is ineffective, and may need higher concentrations. Although it has been suggested that MTT may not be a good estimate for screening natural compounds [30], our controls groups have enabled us to prevent the inclusion of false positive results.

### 2.2. Comparison of Apoptosis Inducing Activity between Types of Fucoidan Combined with GroA

Next, we wanted to examine whether the combined effect of Fucoidan and GroA can increase cell death in the two prostate cancer cell lines. The cells were treated with either GroA or LMWF alone, or with both drugs in combination. Following treatment with each drug alone, the optimal incubation time chosen for the combined treatments was 72 h. At this time point, each drug exerted a significant inhibitory effect on cell growth. Flow cytometry allowed the measurement of several apoptotic traits in a single sample, making it a powerful tool to study the complexity of cell death. Each experiment was performed in triplicate.

DU-145 cells were treated with LMWF alone or in combination with GroA (Supplement Figure S7). Following GroA treatment, the distribution of the apoptotic cells showed that the percentage of viable cells decreased by about 30%, whereas the percentage of early apoptotic cells and late apoptotic cells increased by 23% and 5%, respectively. The cell population of DU-145 treated with different concentrations of LMWF (100, 200 and 300 μg/mL) resulted in the reduction of cell viability (from 97.08 to 77.76%, 68.32% and 51.43%), compared to the control. In addition, the percentages of both early and late apoptotic cells were increased with the increasing concentration of LMWF in the combination group. Treatment with GroA alone compared to the combined treatment with LMWF has shown an increase in both early and late apoptotic population (combined early and late apoptotic cells increased from 28 to 31%, 50% and 58%), and the percentage of viable cells was reduced with increased concentrations of LMWF. Therefore, treatment with a combination of LMWF with GroA caused a significant change in the cell viability and increased apoptosis in the treated DU-145 cells.

The apoptotic cell death observed in treated PC-3 cells was similar to that of DU-145 cells after 72 h (Supplement Figure S8). GroA increased the apoptotic cell death of PC-3 cells compared to control (Cro). LMWF in increasing concentrations (100, 200, and 300 μg/mL) has also elevated the apoptotic cell death. The total apoptotic percentage increased with the increasing concentration of LMWF, and LMWF induced cellular apoptosis increased 20% compared with Cro alone. Moreover, compared to GroA-alone treated cells, the combined treatment of LMWF (300 μg/mL) with GroA raised the apoptotic cell death, with apoptotic cells’ percentage 35% higher than cells treated with GroA alone. Other groups of combined treatments of GroA with different LMWF concentrations also showed remarkable changes, but the combination of 300 μg/mL LMWF with GroA was the most effective in increasing apoptotic cell death. Therefore, treatments with LMWF (100, 200, and 300 μg/mL) resulted in an approximate 20% of cell death of PC-3 and DU-145 cells. Nevertheless, the apoptotic cell death was robust when the cells were treated with the combination of LMWF (100, 200, and 300 μg/mL) and GroA, compared to each drug alone in both cell lines. Thus, the results strongly suggest that the combined treatment of LMWF and GroA can inhibit cell viability, while enhancing apoptotic cell death.

In the combined treatment of FS with GroA, it was found that the distribution of the PC-3 and DU-145 cellular apoptosis characters was only changed by GroA treatment. When cells were treated with FS (500, 750 and 1000 µg/mL) combined with Cro (10 µM), there was no significant effect on cell death compared to Cro-alone treated cells. Furthermore, both GroA and the combination of GroA with FS had lower apoptosis level compared with FS alone, however, there was no statistical significance between GroA and the combination of GroA with FS treatments. Thus, FS alone or in combination with GroA did not induce PC-3 or DU-145 cell apoptosis (data not shown, further experiments only continued working with LMWF due to its effectiveness).

The results demonstrate that the combined effect of LMWF and GroA on DU-145 and PC-3 cell viability and cell death is significantly more effective compared to each of the treatments alone. However, in the results of GroA combined with FS, the combination effect of the two drugs was not improved synergistically. In all the FS concentrations that were tested, it did not increase the inhibitory effect of GroA. There were no significant differences between the cell viability values when cells were treated with 10 µM GroA with or without FS (500, 750 and 1000 µg/mL). The sulfate content of the extracts may provide an explanation for some of the observed results. In a previous study of Fucoidan sulfate content, LMWF sulfate levels were higher than high-molecular-weight Fucoidan, and it was shown that the antiproliferative activity of LMWF was dependent on it, as the antiproliferative effect was reduced with the decreasing degree of sulfation [31]. Furthermore, the differences in Fucoidan structures may help to explain their antiproliferative activities. There are considerable variations in the anticancer activities between Fucoidan polymers, which are isolated from different parts of the same species of source material. For example, the anatomical regions and growing conditions of brown seaweeds, their extraction and purification procedures, as well as the different cancer cell lines that are being tested. All these reasons can explain the observed effects of the treatments in this study.

### 2.3. Alterations of Cell Cycle on LMWF Combined with GroA

**Figure 3:**
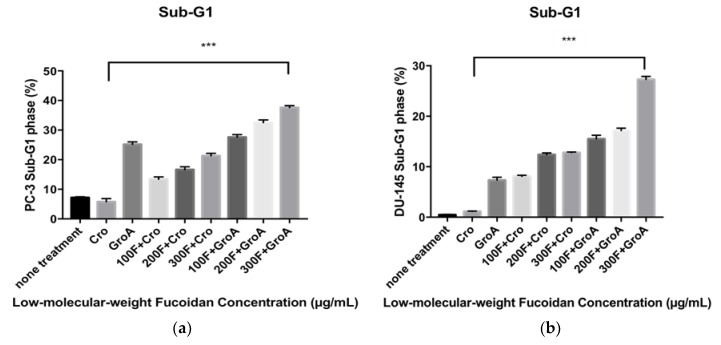
Co-treatment with LMWF and GroA increases the sub-G1 population of PC-3 cells (**a**) and DU-145 (**b**). Cells were treated with LMWF (100, 200 and 300 µg/mL) in the presence or in the absence of 10 µM GroA for 72 h. The percentage of the sub-G1 population is indicated. Data is presented as means ± S.D., n = 6. Asterisks indicate a value significantly different, *** *p* < 0.001 (Student’s *t*-test).

Next, we evaluated the alterations in the cell cycle of the cancer cells following LMWF and GroA treatments by using Flow cytometry analysis with PI DNA staining. The proportions of the sub-G1 hypodiploid cells (which indicate the proportion of cell death) was estimated after 72 h of treatment for each cell line. By comparing the percentage (%) of sub-G1 fractions obtained after cells were treated with different concentrations (100, 200, and 300 µg/mL) of LMWF alone or in combination with Cro/GroA, the effectiveness of the drugs to increase cell death in different combination groups was estimated. Along with determining the index of the apoptotic DNA fragmentation (i.e., % of sub-G1) this procedure helps with simultaneous analysis of cell-cycle parameters of surviving cells (i.e., cell cycle distribution). Thus, any arrest or blockage of cells at any particular cell phase was also examined. Before starting the evaluation of apoptosis inducing activity by different Fucoidan preparations in this study, the effect of serum starvation on each cell line was examined. The sub-G1 population was used as an index of apoptotic DNA fragmentation [32]. LMWF was found to increase the sub-G1 cell population in a dose-dependent manner, while it decreased the G0-G1 population in both PC-3 and DU-145 cell lines (Figure 3). LMWF combined with GroA augmented the induction of apoptotic cell death in both PC-3 (Supplement Figure S9) and DU-145 (Supplement Figure S10) cell lines by increasing the sub-G1 population up to 37.61% at the highest concentration of LMWF. The percentages of sub-G1 populations were higher in all the combined treatments compared to each of the treatments alone. The combination of LMWF and GroA therefore resulted in a synergistic effect. Both GroA and LMWF blocked the sub-G1 phase of the cell cycle distributions measured, despite the fact that the effects of each drug on cellular metabolism are different. Furthermore, the accumulations of G0-G1 phase cells decreased dramatically to 37.31% in cells treated with a concentration of 300 µg/mL LMWF.

Therefore, LMWF has a strong effect on the cell cycle in both prostate cancer cell lines tested by increasing the sub-G1 phase population and decreasing the G0–G1 population. When combined with GroA, a synergistic effect is observed, as LMWF has an even more robust effect on the sub-G1 population, while it also decreases the G0–G1.

### 2.4. Alterations of Caspase-3/7 Activity Resulting from Combined LMWF and GroA Treatment

**Figure 4:**
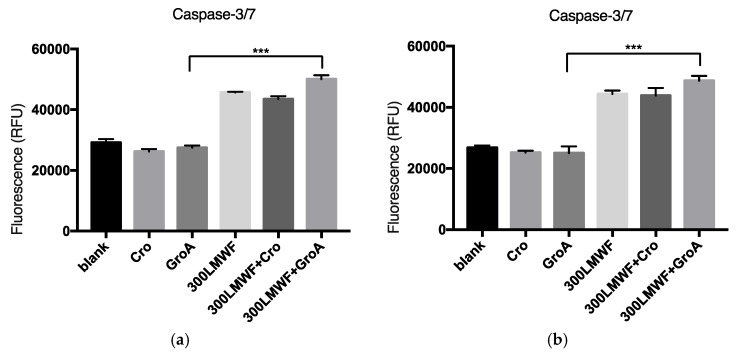
The alterations of caspase-3/7 activity following LMWF (300 µg/mL) and GroA treatments. DU-145 (**a**) and PC-3 (**b**) cell lines were treated for 72 h with LMWF (300 µg/mL) in the presence or in the absence of 10 µM GroA. The cells were tested for Caspase dependent cell apoptosis using the Apo-ONE^®^ Homogeneous Caspase-3/7 Assay. Data is presented as means ± S.D., n = 6. Asterisks indicate a value significantly different, *** *p* < 0.001 (Student’s *t*-test).

To further investigate cell death, we examined whether it is a Caspase dependent. The members of the cysteine aspartic acid-specific protease (Caspase) family play key roles in apoptosis in mammalian cells [33,34,35,36]. The Caspase-3 protein is a member of the cysteine-aspartic acid protease (Caspase) family [37]. The alterations of Caspase-3/7 activity due to LMWF and GroA treatments were evaluated by using Apo-ONE^®^ Homogeneous Caspase-3/7 Reagent (In Vitro Technology, Madison, MI, USA). The Apo-ONE^®^ Homogeneous Caspase-3/7 Assay provides a profluorescent substrate with an optimized bifunctional cell lysis/activity buffer for caspase-3/7 (DEVDase) activity assays. The levels of Caspase-3/7 activity were estimated after the cells were treated with 300 µg/mL of LMWF or in combination with GroA/Cro for 72 h of treatment, and the effectiveness of Caspase-3/7 for different combination groups was estimated. In the present study, LMWF was found to increase the levels of Caspase-3/7 activity in both PC-3 and DU-145 cell lines (Figure 4). GroA by itself did not increase Caspase-3/7 levels, but when combined with LMWF, it elevated the LMWF effect. These results may suggest that LMWF regulates sequential activation of apoptotic cell death, and that at least part of the cell death observed by LMWF and GroA is Caspase-dependent.

### 2.5. Comparison of Results with Past Findings

There are relatively few reports about cell cycle effects and apoptosis in Fucoidan studies. Literature suggests that Fucoidan treatments result in sub G0/G1 cell accumulation (suggestive of dead cells/apoptotic cells) in a variety of cell types [38,39]. It can also induce cell cycle arrest in other phases. Riou et al. [40] and Mourea et al. [41] reported cell arrest in the G1 phase in a chemo-resistant non-small-cell bronchopulmonary carcinoma line by Fucoidan from *Ascophyllum nodosum* and *Bifurcaria bifurcate*, respectively. In an investigation of the mechanism of the action, Fucoidan demonstrated significant down regulation of cyclin D1, cyclin D2, and CDK4 in MCF-7, PC-3, and T-cell leukemia cancer cells [42,43,44]. The crude Fucoidan from *Fucus vesiculosus* increased the levels of p21/WAF1/CIP1 in PC-3 cells and down-regulated E2F, a transcription factor that controls progression of cells from G1 to S phase [43]. An animal study showed that fucoidan significantly hindered the tumor growth and inhibited angiogenesis in DU-145 cell line, with decreased hemoglobin content and reduced mRNA expression of CD31 and CD105 in tumor tissue [45]. Choo et al. [46] also suggest that Fucoidan’s anticancer effect through inhibition of PI3K/Akt and MAPK Pathway Expression in DU-145 prostate cancer cell line.

Our results indicated that LMWF reduced cell growth while it increased cell death, which partially involves apoptotic cell death, in two lines of prostate cancer cell lines (PC-3 and DU-145). The same result was found in DU-145 cells, but LMWF 100 and 200 µg/mL treatments only induced minor sub-G1 effects on DU-145 cells, while 1000 µg/mL led to more sub-G1 changes (an increase of about 27.03%). LMWF was found to decrease the G0–G1 phase in PC-3 cells after 72 h of treatment, with a slight decrease in the phase population as LMWF concentrations increased. LMWF did not show an effect on the S phase and G2-M phase populations compared with the Cro treatment group. LMWF induced apoptosis of PC-3 and DU-145 cells in a time- and dose-dependent manner. Taken together, the current results in PC-3 cells are consistent with previous reports, namely Fucoidan raises the sub-G1 population and the cell line undergoes apoptosis.

## 3. Materials and Methods

### 3.1. Materials

Fucoidan was extracted from *U. pinnatifida* as previously described [47]. Low-molecular-weight Fucoidan (under 10 kDa) was obtained via dialysis using molecular weight cut bags. *U. pinnatifida* Fucoidan (≥95%, CAS: 9072-19-9, from North America) was purchased from Sigma Aldrich (St. Louis, MO, USA). Gro (GroA/AS1411) (10 μmol DNA Oligo, 26 bases) and Cro (10 μmol DNA Oligo, 26 bases) were purchased from Integration DNA Technology (Jerusalem, Israel). PC-3, Human prostate cells, derived from a metastatic site in bone (Cat No. CRL-1435) and DU-145, Human prostate cells, derived from a metastatic site in brain (Cat No. HTB-81) were purchased from American type culture collection (ATCC) (Manassas, VA, USA). Dead Cell Apoptosis Kit with Annexin V FITC and PI (Cat No. V13242) were purchased from Thermo Fisher Scientific (Waltham, MA, US). Ribonuclease A from bovine pancreas (Cat No. R4875—100 mg) and Triton™ X-100 for molecular biology (Cat No. T8787—250ML) were purchased from Sigma-Aldrich (St. Louis, MO, USA). Apo-ONE^®^ Homogeneous Caspase-3/7 Assay 100 mL (Cat No. G7791) was purchased from In Vitro Technology (Madison, WI, USA).

### 3.2. Cell Proliferation Assay

FS was dissolved in cell culture medium (RPMI 1640 base medium with 1% Penicillin-Streptomycin, 1% l-glutamine and 10% fetal bovine serum) to a final concentration of 2000 µg/mL as a stock solution. LMWF from *U. pinnatifida* was extracted at Auckland University of Technology, New Zealand, and dissolved in cell culture medium (RPMI 1640 base medium with 1% Penicillin-Streptomycin, 1% l-glutamine and 10% fetal bovine serum) to a final concentration of 3200 µg/mL and 600 µg/mL as stock solutions. Aliquots of Fucoidan stock solutions were separated into micro-tubes. The micro-tubes were wrapped in aluminum foil and stored at −80 °C. The aptamer Gro, and the inactive oligomer Cro, were dissolved in double distilled water (DDW) to a final concentration of 1000 μM as stock solutions, and incubated at 65 °C for 15 min. Aliquots of GroA stock solution were separated into micro-tubes and stored at −20 °C. Basic cell culture techniques were carried out prior to and during experiments. Cells were seeded (50,000 cells/mL), 100 μL in each well in 96-well plates, as required. Treatments, 100 μL per well, were incubated for a set time (normally 72 and 96 h), replacing the original medium with 100 μL of fresh culture medium. Then, 10 μL MTT stock solution was added prior to incubating at 37 °C. An aliquot of DMSO (100 μL) was added to each well and mixed thoroughly using an orbital plate shaker. After incubating at 37 °C for 20 to 30 min, the plate was shaken briefly and absorbance was measured by a plate reader (FLUOstar Omega, Alphatech) at a wavelength of 540 nm, with the reference wavelength at 680 nm. The average absorbance value (OD value) was determined from sextuplicate readings, and the average values were subtracted from the average value from the blank readings in order to determine the final absorbance value.

All the concentrations of FS (500, 750, and 1000 μg/mL), LMWF (100, 200, and 300 μg/mL) and GroA (AS1411)/Cro 10 µM were used to treat PC-3 and DU-145. Controls (including blank, cells only, fucoidan only, and none treatment groups) were added to ensure false positive results were eliminated.

### 3.3. Cell Apoptosis Assay

The FITC Annexin V/Dead Cell Apoptosis Kit with FITC Annexin V and PI for flow cytometry provides a rapid and convenient assay for apoptosis. The kit contains recombinant annexin V conjugated to fluorescein (FITC annexin V), as well as a ready-to-use solution of the red-fluorescent propidium iodide (PI), a nucleic acid binding dye. Cells were seeded onto a 6-well plate containing 40,000 cell/mL, and a 2 mL cell solution was contained in each well. The plates were kept at 37 °C for 6 to 24 h to ensure almost all of the cells attached to the wells. All treatments were kept in 15 mL centrifuge tubes which were prepared with complete culture (10% FBS) medium. The spent complete culture medium was removed gently and 2 mL well-mixed treatment solution was carefully added into designated wells. The control well was set in the plate of the treatment group with 2 mL complete culture medium. All of the plates were kept in the incubator for 72 h.

After completing their time course, cells were harvested and stored for analysis. Appropriate washing steps were performed and then trypsin treatment was prepared cells for Annexin assays. Cells were re-suspended in 1X annexin-binding buffer. Cell density was determined and diluted in 1X annexin-binding buffer to ~1 × 10^6^ cells/mL, preparing a sufficient volume to have 100 μL per assay. Reagents were added according to manufacturer’s protocol, and stained cells were analyzed by flow cytometry, measuring the fluorescence emission at 530 nm (e.g., FL1) and >575 nm (e.g., FL3). Flow cytometry results were confirmed by fluorescence microscopy, using filters appropriate for fluorescein (FITC) and rhodamine (TRITC) or Texas Red^®^ dye.

### 3.4. Cell Cycle Assay

All cell lines in this study were seeded at 40,000 cells/mL in a 2 mL cell solution in each well. The plates were kept at 37 °C for 6 to 24 h. When the cell attachment rate was the highest, cells were treated with 2 mL of 0% FBS medium (with 1% Penicillin and 1% l-glutamine) in each well in order to synchronize cell proliferation. All treatments were kept in 15 mL centrifuge tubes and were prepared with complete culture (10% FBS) medium. The old 0% FBS medium was removed gently, and then 2 mL well-mixed treatment solution was carefully added into designated wells.

After cells were cultured for their determined time, they were harvested and stored for analysis. Appropriate washing and trypsinization prepared cells for subsequent manipulations. After centrifuging, the supernatant in each tube was discarded. One milliliter of cold 80% ethanol was added into each tube. (Each tube was previously stored in the −20 °C freezer). The vortex was set at a low speed, and ethanol was added slowly. The tube was tilted diagonally so the ethanol was added to the sides and not directly onto the cells, thereby avoiding the formation of aggregates. Each tube was sealed with parafilm and kept at −20 °C for at least overnight and not more than 10–14 days.

A permeabilizing solution was prepared and added to samples as per the manufacturer’s recommendations. All tubes were centrifuged first at 1200 RPM for 2 min after removing the parafilm from the outside of the tubes. The ethanol was gently removed, and 3 mL ice cold PBS was added to each tube, followed by a second centrifugation step. After the second centrifugation, another 3 mL ice cold PBS was added to each tube to replace the initial wash. In total, the cells were washed twice, each time with 3 mL ice cold PBS. In the process of PI staining, the supernatant in each well was gently removed, and then 1 mL well-mixed permeabilizing solution was added to each tube. All cells in tubes were carefully mixed before being transferred to test tubes and then incubated at 37 °C for 30–45 min. After permeabilization, 5 μg/mL of PI (5 μL to 1 mL in each tube) was added to each test tube and kept for 5 min. Finally, all tubes were run under the flow cytometer.

### 3.5. The Apo-ONE^®^ Homogeneous Caspase-3/7 Assay

Reagents were prepared according to the manufacturer’s recommendations. Cells were seeded on 96-well plates (50,000 cells/mL), 50 μL each well. Treatments, 50 μL to each well, were added and incubated for a set time (normally 72 and 96 h). Apo-ONE^®^ Caspase-3/7 Reagent was added to blank, control or cells in culture. Samples were carried out in triplicate. Plates were incubated on a shaker (300–500 rpm) at room temperature for 30 min to 18 h depending upon expected level of apoptosis (and thus, caspase-3/7 activity) in the cells analyzed. Fluorescence of each well was measured at the optimal excitation wavelength of 499 nm with the emission maximum at a wavelength of 521 nm.

### 3.6. Statistical Analysis

Statistical analysis was performed by using GraphPad Prism (GraphPad Software, San Diego, CA, USA) software package. Results were expressed as mean ± SEM (sample n = 3 with triplicate analysis done on each sample). Analyses of variance were performed using one-way ANOVA, with post hoc Tuckey’s test for significant differences. A nominal two-sided *p* < 0.05 was used to assess significance.

## 4. Conclusions

The present study describes the effect of LMWF, FS, or combinations of either drug with GroA, on cell proliferation and cell death of human prostate cancer cell lines PC-3 and DU-145. It was shown that LMWF inhibited the growth and viability of the cancer cells in a time- and dose-dependent manner. Combination of LMWF and GroA had a synergistic inhibitory effect on the growth and viability of the prostate cancer cells, hence we consider it as a potential candidate for the treatment of prostate cancer. On the other hand, the effect of FS was not significant at the concentrations and combinations tested. In the combined treatment of FS and GroA, FS was not able to enhance the inhibitory effect of GroA on cell proliferation of PC-3 and DU-145 cells, even at very high concentrations.

We found that even at low doses, LMWF significantly improved the anti-proliferative effect of GroA. Furthermore, the combination of LMWF and GroA not only induces cell growth inhibition, but it also enhanced cell death induced by each of the drugs alone and by the combined treatment. Moreover, at least part of the cell death induced is a caspase-dependent apoptotic cell death. In summary, this study shows that LMWF can synergistically increase GroA’s anti-cancer effect in the tested cell lines, and together they induce a stronger inhibitory effect on cancer cell growth and tumorogenicity. This indicates that LMWF has the potential to be developed into a supplement therapy for prostate cancer.

## Supplementary Materials

The following are available online at http://www.mdpi.com/1660-3397/16/11/454/s1.
