# Supplementary material for: Investigation of Different Molecular Weight Fucoidan Fractions Derived from New Zealand Undaria pinnatifida in Combination with GroA Therapy in Prostate Cancer Cell Lines
Source: Mar Drugs. 2018 Nov 18;16(11):454. doi: 10.3390/md16110454 (PMC6266598; doi:10.3390/md16110454)
Supplement: Supplementary file 1 [file marinedrugs-16-00454-s001.docx]

**Supplement Figure 1. The inhibitory effect of FS compared with LMWF on cell growth and viability in PC-3 cells.** Cells were incubated in the presence of various concentrations of LMWF (100, 200 and 300 µg/ml), FS (500, 750 and 1000 µg/ml). A relative cell viability of 100% was designated as the total number of cells that grew after 72 and 96 hours cultures in the absence of LMWF and FS. Data is presented as means ± S.D., n=6. Asterisks indicate a value significantly different from the control value, ***p < 0.001 (Student’s t-test).

**Supplement Figure 2.** The inhibitory effect of FS compared with LMWF on cell growth and viability in DU-145 cells. Cells were incubated in the presence of various concentrations of LMWF (100, 200 and 300 µg/ml), FS (500, 750 and 1000 µg/ml). A relative cell viability of 100% was designated as the total number of cells that grew after 72 and 96 hours cultures in the absence of LMWF and FS. Data is presented as means ± S.D., n=6. Asterisks indicate a value significantly different from the control value, **p < 0.01, ***p < 0.001 (Student’s t-test).

**Supplement Figure 3. The combined inhibitory effect of** **GroA/Cro and LMWF on the growth of PC-3 cells.** Cells were incubated in the presence of various concentrations of LMWF (100, 200 and 300 µg/ml), GroA/Cro (10 µM), and compare with combination treatment (LMWF+GroA/Cro). A relative cell viability of 100% was designated as the total number of cells that grew after 72 and 96 hours cultures in the absence of LMWF and GroA/Cro. Data is presented as means ± S.D., n=6. Asterisks indicate a value significantly different, ***p < 0.001 (Student’s t-test).

**Supplement Figure 4. The Combined inhibitory effect of GroA/Cro and LMWF on the growth of DU-145 cells**. Cells were incubated in the presence of various concentrations of LMWF (100, 200 and 300 µg/ml), GroA/Cro (10 µM), and compare with combination treatments (LMWF+GroA/Cro). A relative cell viability of 100% was designated as the total number of cells that grew after 72 and 96 hours cultures in the absence of LMWF and GroA/Cro. Data is presented as means ± S.D., n=6. Asterisks indicate a value significantly different, ***p < 0.001 (Student’s t-test).

**Supplement Figure 5. Combined inhibitory effect of GroA/Cro and FS on the growth of PC-3 cells.** Cells were incubated in the presence of various concentrations of FS (500, 750 and 1000 µg/ml), GroA/Cro (10 µM), and compare with combination treatments (FS+GroA/Cro) for 72- and 96- hours. A relative cell viability of 100% was designated as the total number of cells that grew after 72- and 96-hours cultures in the absence of FS and GroA/Cro. Data is presented as means ± S.D., n=6.

**Supplement Figure 6. The Combined inhibitory effect of GroA/Cro and FS on the cell growth and viability in DU-145 cells.** Cells were incubated in the presence of various concentrations of FS (500, 750 and 1000 µg/ml), GroA/Cro (10 µM), and compare with combination treatment (FS+GroA/Cro) for 72- and 96- hours. A relative cell viability of 100% was designated as the total number of cells that grew after 72 and 96 hours cultures in the absence of FS and GroA/Cro. Data is presented as means ± S.D., n=6.

**(A)** Control group


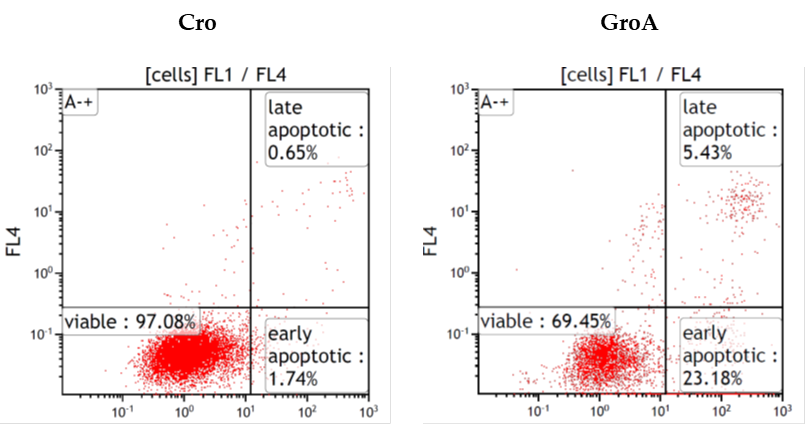


**(B)** Combined treatment group

**Supplement Figure 7. The combined treatment of GroA and LMWF** **increases apoptosis in DU-145 cells**. Cells were incubated with various concentrations LMWF (100, 200 and 300 µg/ml), in the presence or in the absence of 10 µM GroA/Cro for 72 hours. (A) Control group, treated with 10 µM Cro and GroA. (B) Combined treatment groups of LMWF (100, 200 and 300 µg/ml) with GroA/Cro (10 µM).

**(A) Control group**

**(B) Combined treatment group**

**Supplement Figure 8. The combined treatment of GroA and LMWF** **increases apoptosis in PC-3 cells**. Cells were incubated with various concentrations LMWF (100, 200 and 300 µg/ml), in the presence or in the absence of 10 µM GroA/Cro for 72 hours. (A) Control group, treated with 10 µM Cro and GroA. (B) Combined treatment groups of LMWF (100, 200 and 300 µg/ml) with GroA/Cro (10 µM).

**Supplement Figure 9. The Cell cycle distributions of** **PC-3 cells following treatments with either GroA or LMWF or both**. Cells were treated for 72 hours with 10 µM GroA /Cro in the presence and in the absence of LMWF (100, 200 and 300 µg/ml). The cells were then harvested and analyzed for their DNA content by flow cytometry. The percentage of live cells at different cell cycle stages is indicated.

**Supplement Figure 10. The cell cycle distributions of DU-145 cells following treatments with either GroA or LMWF alone both.** Cells were treated for 72 hours with 10 µM GroA/Cro in the presence and in the absence of LMWF (100, 200 and 300 µg/ml). The cells were then harvested and analyzed for their DNA content by flow cytometry. The percentage of live cells at different cell cycle stages is indicated.
